# ChatGPT in Dentistry: A Comprehensive Review

**DOI:** 10.7759/cureus.38317

**Published:** 2023-04-30

**Authors:** Hind M Alhaidry, Bader Fatani, Jenan O Alrayes, Aljowhara M Almana, Nawaf K Alfhaed

**Affiliations:** 1 Advanced General Dentistry, Prince Sultan Military Medical City, Riyadh, SAU; 2 Dentistry, College of Dentistry, King Saud University, Riyadh, SAU

**Keywords:** healthcare, artificial intelligence, dental, dentistry, chatgpt

## Abstract

Chat generative pre-trained transformer (ChatGPT) is an artificial intelligence chatbot that uses natural language processing that can respond to human input in a conversational manner. ChatGPT has numerous applications in the health care system including dentistry; it is used in diagnoses and for assessing disease risk and scheduling appointments. It also has a role in scientific research. In the dental field, it has provided many benefits such as detecting dental and maxillofacial abnormalities on panoramic radiographs and identifying different dental restorations. Therefore, it helps in decreasing the workload. But even with these benefits, one should take into consideration the risks and limitations of this chatbot. Few articles mentioned the use of ChatGPT in dentistry. This comprehensive review represents data collected from 66 relevant articles using PubMed and Google Scholar as databases. This review aims to discuss all relevant published articles on the use of ChatGPT in dentistry.

## Introduction and background

Chat generative pre-trained transformer (ChatGPT) is an artificial intelligence (AI)-based computer program that has been trained on enormous amounts of data to produce responses to user prompts that are human-like to improve the computational linguistics, communication competence, and responsiveness of these bots. Techniques like machine learning and deep learning are used via text-based interfaces [1-4]. ChatGPT uses deep learning AI techniques to produce human-like responses to natural language queries, making it a very large language model [5]. ChatGPT can provide multiple services for education, healthcare providers, and even patients. For instance, for students, it could offer schoolwork guidance and tutoring by responding to inquiries and outlining details to make complex ideas clear to them, and by acting as a teaching tool, it has the potential to completely change how students learn biomedical science [6,7]. In dentistry and healthcare, ChatGPT can offer multiple services for healthcare personnel, including better diagnosis, supporting decision-making, digital data recording, analyzing images, preventing diseases, treating diseases, reducing the number of treatment mishaps, and enabling exploration and research [3,4,8]. ChatGPT is extremely helpful for patients as it can answer medical questions, especially for patients undergoing a surgical procedure, as it can aid in educating the patient pre- and post-surgery and provide a reasonable expectation for the outcome of the surgery [2,9]. These applications of ChatGPT should show a significant benefit in health care and dentistry by enhancing patient empowerment and independence, raising service effectiveness and safety, enhancing sustainability, expanding access to and quality of care, or empowering and enabling patients [8].

## Review

Methods

This study comprehensively reviewed published articles that focused on the utilization of ChatGPT in the dental and health care fields. The population, intervention, control, and outcomes (PICO) framework was used to determine whether ChatGPT is beneficial or not as follows: in dentistry and other health care specialties, what is the benefit of ChatGPT compared to other methods? PubMed and Google Scholar were among the various databases used to collect relevant articles. A search set combined a range of keywords such as "medicine", "dentistry", and "ChatGPT" to gather all the relevant papers on this topic from PubMed and Google Scholar. By using this approach, all articles related to ChatGPT's application in dental and medical areas were retrieved, and after the screening, 66 relevant papers were reviewed. Overall, this study analyzed the usefulness of ChatGPT in health care and dentistry by considering various published articles.

Artificial intelligence

AI can be defined as a series of actions intended to carry out a particular task [10]. It is a branch of computer science that has the potential to imitate human intellect in order to make predictions and make complicated decisions [10,11]. It is starting to influence a variety of conveniences such as content recommendation, face recognition, speakers with built-in AI, and more [12,13]. Hence, in recent years, AI has been applied in a wide range of specialties and opened a wide range of exciting opportunities. A major development has been made in dentistry and the medical field when it comes to AI, including medical image analysis, data mining, and natural language processing [12]. With advancements in high-performance computing, it has been possible to extract and acquire the required data from the pool of collected data. This process of information extraction is referred to as machine learning (ML) [13]. ML is a subtype of AI, and instead of being explicitly coded with rules and assertions, ML learns to complete tasks by identifying patterns in data. On the other hand, a specific type of machine learning known as deep learning (DL) is based on artificial neural networks, which were inspired by the human nervous system. The network architecture of the model is said to be "deep" because it has several layers. Traditional ML involves the model learning from manually derived data features, whereas DL involves learning from raw input data, including feature extraction. Therefore, DL can outperform ML when it comes to performing complex tasks [14].

ChatGPT in health care

From the standpoint of medical practice, ChatGPT has a huge impact on the medical field and the health care system [8]. ChatGPT can be used as a supplementary tool for diagnosing and making decisions in multiple fields, including cardiology, radiology, and urology [15]. However, questions can be raised regarding the generated output's accuracy and the potential to reinforce diagnoses with biases. This is why human supervision is important when using it [15,16]. Additionally, it is also used for assessing disease risk and consequences, drug development, increasing biomedical research, and having the potential to revolutionize health care practice [16]. Recently, ChatGPT demonstrated the capability to generate effective discharge summaries, which can be useful to lessen the burden of documentation in the health care industry [8]. In addition, ChatGPT has the potential to assist in the efficiency of service delivery by optimizing clinical workflow and reducing costs [16]. ChatGPT even showed moderate accuracy in breast cancer screening, assessing pain, and identifying the imaging steps required [15]. Nevertheless, ChatGPT has some drawbacks as with any new development, including a hugely expensive cost, a large-scale neural network that requires substantial computational power and considerable memory, making it more concerning to utilize and integrate into smaller medical applications, and the inability of ChatGPT to integrate external information sources, which limits the accuracy of the system in the medical industry [15]. Additionally, the inability of the proposed method, ChatGPT, to integrate external information sources such as textbooks or medical journals is why it restricts its precision in the medical industry. Also, ChatGPT's responses may be unreliable, resulting in misunderstandings and a loss of trust in the medical community [15]. Graduate medical education needs to factor in various aspects to ensure that AI is ethically applied in clinical care and educational processes. The increasing use of this technology necessitates a prompt assessment of program procedures, policy updates, and a significant national debate [17]. 

Gupta et al. explained that plastic surgeons should use ChatGPT, a versatile tool with benefits beyond research, such as patient consultation, support, and marketing [18]. With the ongoing advances in AI, it is significant for plastic surgeons to integrate AI into their clinical practice. AI was also discussed in the psychiatry field by Prada et al. [19]. ChatGPT has been successful in many areas of study, even though it has not received specialized training in those areas. It has demonstrated its ability to perform at or near the level of post-graduate exams in a variety of fields [20]. ChatGPT is capable of solving complex reasoning questions in pathology with a high degree of accuracy. As a result, it produces text output with connected parts that provide a meaningful response. This level of AI cognition can assist students and academics in obtaining useful answers to their inquiries [21]. AI models such as ChatGPT can also aid the health care sector significantly by offering a more impartial and fact-driven decision-making process and lowering the chances of mistakes by utilizing their unmatched data-processing speed. It can also recognize trends and connections in extensive data sets, which can lead to innovative health care insights and progress. Moreover, AI models can identify diseases and anticipate prognosis, enabling more tailored therapy suggestions and advancing patient outcomes [22]. The health care industry should embrace the use of AI to improve the efficiency of physicians and reduce their reliance on computers, rather than waiting for AI algorithms to independently learn how to read CT scans or diagnose medical conditions [23].

Chatbot technology like GPT could bring a significant transformation to medical writing, enhancing the writing process's effectiveness by automating some tasks [24]. This AI-powered chatbot could be used to aid in scientific writing by creating automatic drafts, summarizing articles, and translating content from different languages. Utilizing this technology could speed up academic writing and make it easier. However, ethical concerns arise, and therefore its usage in scientific writing should be closely monitored and regulated [25]. Using ChatGPT for language editing in scientific articles is acceptable, but any novel concepts created by ChatGPT must undergo empirical testing and be reviewed by humans [26]. Researchers and practitioners can make use of ChatGPT in a judicious manner by obtaining a precise comprehension of its abilities and limitations, thereby preventing any unwanted consequences. Additionally, the community can recognize areas that require further research and development in order to enhance the performance and capabilities of the model by defining its limitations. So far, there have been many challenges associated with the use of these devices in both clinical care and research due to their considerable restrictions [27]. ChatGPT's ability to generate coherent and grammatically correct text that is indistinguishable from human-written content is noticeable and poses a significant threat to academic publishing methods [28]. Currently, ChatGPT can be used for free via the ChatGPT Research Preview. However, there is a potential issue that ChatGPT and other similar AIs might become too expensive to subscribe to as they gain popularity. This could result in an unequal distribution of resources among researchers in various fields [29]. When using ChatGPT, it is important to keep in mind that it does not always provide accurate references or any at all; answers may vary depending on the question and preceding conversation or context; and it may give incorrect answers that sound convincing, which is a problem that needs to be fixed to ensure that ChatGPT can acknowledge uncertainty appropriately [30]. Lastly, since the production of inaccurate content can have serious negative effects on health care, this legitimate concern should be carefully taken into account in clinical practice, and a valid concern must be obtained [16].

AI in dentistry

Advancements in AI technology have given rise to both aspirations and apprehensions in health care, particularly in the realm of dentistry [31]. The next decade will determine if the high hopes for AI applications are met or if there will be another AI winter. This is particularly important in health care, where there are concerns about data protection and the use of computers to make critical medical decisions. Despite these concerns, AI has the potential to revolutionize health care and improve dental care. Dental research can play a role in ensuring that AI is used to make dental care better, more affordable, and to the benefit of patients, providers, and society as a whole [8]. The emergence of AI in dentistry is transforming the field, allowing for greater precision, fewer errors, and requiring less staffing. AI can perform various tasks in the dental clinic, like scheduling appointments and assisting with clinical diagnosis and treatment planning. AI has already shown high accuracy, sensitivity, specificity, and precision in detecting and classifying malocclusion in orthodontics. Additionally, AI can automatically classify dental restorations on panoramic radiographs and detect dental and maxillofacial abnormalities such as periodontal diseases, root caries, bony lesions, and facial defects [32]. Grischke et al. explained in their paper that dentistry can greatly profit from the current increase in digital automation that's focused on humans, leading to a new era of robots, machine learning, and AI [33]. This new era, called Dentronics, could significantly improve reliability, reproducibility, accuracy, and efficiency in dentistry by making use of modern dental technologies such as medical robots and specialized AI. Dentronics could also increase our comprehension of disease pathogenesis, enhance risk-assessment strategies, diagnoses, and disease prediction, as well as lead to better treatment outcomes [33]. AI is making rapid strides and has the potential to be used in diagnosis, treatment, and prognosis. However, there are obstacles like obtaining data, interpreting it, computing power, and moral concerns that need to be addressed. Despite these challenges, AI is viewed as a useful addition to dental practice. With careful planning and extensive clinical validation, AI can be made user-friendly, transparent, reproducible, and fair [34]. AI is currently under investigation in dentistry for a range of purposes, with a particular focus on identifying normal and abnormal structures, diagnosing diseases, and forecasting treatment results [10]. Moreover, machine learning (ML) has been utilized for various tasks in dentistry, utilizing diverse methodologies and utilizing vastly different reporting metrics [35]. AI can be used by dental professionals as an extra tool to decrease their workload and enhance the precision and accuracy of diagnosis, decision-making, treatment planning, prediction of treatment outcomes, and disease prognosis [36].

Neural networks have various applications in dentistry. In restorative dentistry, they can detect tooth decay or restorations and aid in selecting the appropriate caries excavation method [37]. Kühnisch et al. concluded that using a standardized, single-tooth photograph and the AI method can result in caries detection with over 90% accuracy [38]. In endodontics, they can detect periapical lesions and root fractures, evaluate root canal systems, predict the viability of dental pulp stem cells, determine working length measurements, and forecast the outcome of retreatment procedures. In orthodontics, neural networks can help diagnose and plan treatments, mark cephalometric points, analyze anatomy, assess growth and development, and evaluate treatment results. Lastly, in dental surgery, they can assist in planning orthognathic surgery, predict post-extraction complications, detect bone lesions, and plan dental implant treatments [38]. A previous systematic review reported that the AI models show great potential for recognizing implant types, predicting implant success, and optimizing implant design, but are still in the developmental stage [39]. Moreover, Bernauer et al. explained that the newest advancements in AI for prosthodontics highlight its use as an automated diagnostic tool, a predictive measure, and a tool for classification and identification [40]. AI is commonly utilized in pediatric dentistry to aid clinicians, dentists, and pediatric dentists in developing an accurate diagnosis, making clinical decisions, creating preventive strategies, and devising an effective treatment plan [41]. AI is not fully utilized in periodontology and implantology, and it is still in its early stages. However, its benefits of aiding diagnosis, analyzing data, and performing detailed regression suggest that there is a lot to be gained from utilizing this tool [42].

ChatGPT in dentistry

Strunga et al. explored the potential of advanced AI software in orthodontics, specifically for applications such as CBCT diagnosis and assessment, evaluation of treatment progress, and ensuring the stability of outcomes during the follow-up phase [4]. The author concluded that using AI technology in orthodontics to evaluate and maintain treatment is a new field that has the potential to greatly improve patient care and outcomes. It is expected that more AI-powered tools and systems will be created and adopted in orthodontics in the future [4]. However, it is important for orthodontists to be properly trained and involved in the use of these systems for the best results. Unsupervised use of AI-assisted systems is not recommended and goes against medical ethics. Current limitations of AI-powered systems in orthodontics include accuracy, expertise, ethical concerns, cost, and regulatory issues. Nonetheless, AI-powered systems have already had an impact on modern orthodontic practice [4]. AI algorithms can be trained to analyze CBCT images for dental conditions by identifying the alignment, location of teeth, and assessing bone quality for implant placement, thus leading to AI-assisted image processing and analysis [43].

Balel et al. evaluated the use of the generated information by ChatGPT in the field of oral and maxillofacial surgery [2]. The author explained that ChatGPT shows promise as a means of providing patient information for oral and maxillofacial surgery. Nonetheless, its use in training is not yet totally secure. Therefore, surgeons should be careful when using ChatGPT and view it as an addition to their practical knowledge and experience [2]. AI has demonstrated precise forecasting of rhinoplasty results and the future need for orthognathic surgery in cleft patients. Nevertheless, it is crucial to enhance current models by examining diverse datasets, surgical approaches, and various populations and surgeries to attain ideal and versatile outcomes [44]. Vinayahalingam et al. reported that the automated segmentation tool that uses AI accurately, quickly, and consistently segmented the mandibular condyles and glenoid fossae [45]. Moreover, Heo et al. explained that oral and maxillofacial radiologists, being experts in radiographic imaging, will have a significant role to play in the advancement of AI applications within their industry [12]. A previous study has shown that the use of AI can improve diagnosis and treatment, resulting in better endodontic treatment outcomes. However, it is crucial to confirm the reliability, applicability, and cost-effectiveness of AI models before implementing them into daily clinical practice [11]. AI has been shown to be accurate in diagnosing and predicting outcomes in endodontics. Its use can improve treatment plans, resulting in higher success rates. Endodontics relies heavily on AI for clinical applications, including detecting root fractures and periapical pathologies, determining working lengths, tracing the apical foramen, identifying root morphology, and predicting diseases [46]. In addition, more and more research is being done on the use of AI and machine learning to help identify head and neck cancer through various imaging methods. These techniques are capable of achieving a level of accuracy that surpasses that of human judgment when it comes to predicting data [47]. Moreover, deep learning methods have enhanced diagnostic procedures in dental radiology by surpassing the accuracy and efficiency of clinicians. They allow for a reduction in the time spent on tasks, a decrease in missed findings, and the prevention of overtreatment [48].

Advanced language models, such as ChatGPT, have immense potential to enhance clinical applications and research in dentistry. Their judicious utilization can bring about a revolution in dental diagnosis and treatment planning. Additionally, investigations based on diverse medical examination data can aid in achieving the objectives of precision medicine and personalized medicine in dentistry. Moreover, the use of multi-modal large language models (LLMs) will also decrease medical expenses and enhance medical efficiency [49]. LLMs are unlikely to have a major effect on dental practitioners, assistants, and hygienists. Eggmann et al. concluded that the potential benefits of utilizing LLMs as supplementary tools in the field of dental medicine are significant [3]. However, it is equally important to take into account the inherent limitations and potential risks involved in implementing such AI technologies. Thus, it is essential to exercise caution and carefully evaluate the advantages and drawbacks before using LLMs in dental practice [3]. Ali et al. assessed how well ChatGPT, an AI-based tool, performs on various dental assessments and explore its impact on undergraduate dental education [50]. The author explained that despite their current limitations, generative AI-based applications can transform virtual learning. Dental educators should adjust teaching and assessments to enhance the learners' benefits while safeguarding against unethical use of AI-based applications [50]. ChatGPT has advantages and disadvantages. While it can benefit students and teachers, it can also generate assignments and answers, leading to academic dishonesty. Although it has some limitations, it can revolutionize virtual learning. Rather than treating it as a threat, dental educators should adjust teaching and assessments to ensure that learners benefit without resorting to academic dishonesty [50]. Moreover, it is important to verify the cost-effectiveness, dependability, and usability of AI models before integrating them into regular clinical operations [51]. ChatGPT can provide patients with reliable and accurate information about dental health and hygiene that caters to their specific needs. It can also serve as an educational resource for students and professionals in the field by providing a vast amount of knowledge and information. Furthermore, ChatGPT can assist dentists with personalized patient care, scheduling, billing, diagnosis, and treatment planning, as well as monitoring patients' dental health and hygiene by providing regular reminders and check-ins [52]. Although ChatGPT is a valuable resource for dental professionals and patients, it is important to acknowledge its limitations. While it can provide accurate information and advice, it cannot provide personalized care, emotional support, or physical treatments. Hence, ChatGPT should be seen as a supplementary tool in dentistry, not a replacement for in-person care. To ensure the best possible outcomes for patients, it is critical to evaluate the pros and cons of using ChatGPT in dentistry and utilize it carefully [53]. Chatbots such as ChatGPT can offer readers immediate feedback, clarify doubts, and provide extra resources. They can also notify readers about newly released publications and make sure that they stay updated on the latest research. The impact of AI and natural language processing technologies like ChatGPT in the publishing process is indisputable, as they allow researchers to communicate their discoveries more effectively, precisely, and comprehensively. By offering clear language, prompt responses, and updated information, ChatGPT has the potential to transform the dissemination and sharing of scientific research [54].

Critical concerns

ChatGPT is a very useful chatbot that can be helpful in many applications in dentistry and health care [3,55]. However, it comes with its own disadvantages and limitations. The use of ChatGPT can place the patient’s privacy at risk because it needs to collect and store the patient’s data and medical history to perform its required task [3]. When it comes to privacy risks, ChatGPT contains personal information from all over the internet [56]. This raises concerns regarding patients’ confidentiality [3]. ChatGPT has greatly impacted dental education and higher education. Hence, students can use ChatGPT to write assignments without fully comprehending them and putting in their full effort. Therefore, traditional efforts to write an essay may need to be changed [7]. People, mostly non-native speakers, have used ChatGPT to make their scientific writing more fluent and to reach standards [3]. But when it comes to writing, ChatGPT has demonstrated flawed and fabricated research and even plagiarism has been noted [3]. Huh et al. have established that when inputting a question in ChatGPT it cannot retrieve data or information from figures, graphs, or tables [6]. Many articles have suggested the incorporation of humans to monitor this technology [3]. Strunga et al. concluded that the application of AI in orthodontics needs supervision by humans, otherwise, it will not meet the standards of health care or medical ethics [4]. ChatGPT does not have the characteristics of human empathy or sympathy. Thereby, when it comes to emotions, ChatGPT cannot replace a human. Hence, the use of it in counseling and therapy is not beneficial [56]. Vishwanathaiah et al. stated that AI can never take the place of a pediatric dentist, but it may be of assistance and complementary aid when it comes to other specialties in dentistry [41]. ChatGPT has some drawbacks despite its advantages. One drawback is that it can produce answers that appear reliable but are incorrect. This is a known issue in many natural language processing models, dubbed the hallucination effect. Additionally, ChatGPT has a tendency to adhere to instructions rather than involving in sincere interaction. For example, when there is insufficient user input, ChatGPT may make assumptions about the desired response without requesting clarification [57]. ChatGPT is useful for people who need basic information on almost any subject. However, since its educational role is still unclear, we should be aware of its limitations [58]. Moreover, the responses produced by ChatGPT were said to be unworthy because they tend to be general, lacking specificity and references [59]. Although AI systems can be highly capable and useful, they are not infallible. Medical practitioners and doctors may become overly reliant on AI systems, potentially leading them to trust the technology's decisions without properly accounting for potential errors and limitations [60]. The major obstacles preventing ChatGPT from being used in clinical settings involve limitations in its ability to understand the situation, make logical deductions, and produce consistent results. These weaknesses could potentially put patients at risk. Despite not having direct access to medical databases, ChatGPT seems to be trained with enough relevant data. Even though it has not been specifically trained in clinical advice, ChatGPT is still able to provide convincing responses to most inquiries [61]. OpenAI acknowledges that ChatGPT may write answers that sound reasonable but are incorrect or nonsensical. Although there is great potential for AI in dentistry, academic and scientific journals may face challenges in identifying whether the text is created by humans or AI in the near future [62]. Some educators are concerned that students might also use ChatGPT to get their work done quickly since it can generate satisfactory text swiftly. This poses a challenge in recognizing cases of plagiarism, which concerns certain educators [63].

Future recommendations

In the upcoming years, AI is highly likely to have a significant effect on several different aspects of the medical and dental fields [2,64]. It can be difficult to imagine the precise scope of these effects. However, AI is still in its early stages, and additional research is required to fully understand its potential benefits [3]. ChatGPT can process information at an unmatched speed and assist the health care field by offering a more objective and evidence-based approach to decision-making and decreasing the chances of human mistakes, and it can also help with disease diagnosis and prognosis and additionally provide a more individualized treatment plan [65]. However, ChatGPT is a double-edged sword, having both strong features and possible drawbacks. Hence, ChatGPT can still produce inaccurate content and yet have detrimental effects on health care such as bias, prejudice, and privacy problems, which should not be taken easily [16,65]. These limitations should be taken into careful consideration when implementing them in clinical practice. Therefore, additional research is required to investigate ChatGPT's effect on quality and efficiency with regard to its assessment tools [16]. When it comes to medical education, ChatGPT requires a high level of accuracy in order to prevent the consequences of errors that may lead to significant harm to the patient’s safety. Although ChatGPT has a substantial amount of data, the possibility of it providing incorrect information may be difficult for students with limited backgrounds to detect. Therefore, clear guidelines and verification procedures should be created and thoroughly tested by responsible personnel. It is a concern that students may rely on ChatGPT as a primary informational source and that it may also facilitate academic dishonesty. This can be prevented by implementing plagiarism technologies that can detect AI content [7]. It is recommended that when starting to adopt AI technologies in the workflow of the health care system, a human should be involved in the loop. Therefore, the output can be monitored and confirmed by human health care providers to minimize the potential risk of error [66].

## Conclusions

AI has shown a great development in dentistry, especially in the field of research as shown in our current review. ChatGPT can potentially transform the dental and healthcare system due to its multiple services such as treatment planning and monitoring individuals' dental health or hygiene. However, caution is still needed, and policies must be developed to reduce the potential hazards. In addition, continuous monitoring of this chatbot is recommended especially in the research field due to multiple concerns regarding ethical issues and improper reference generation.

## References

[1] Gilson A, Safranek CW, Huang T, Socrates V, Chi L, Taylor RA, Chartash D (2023). How does ChatGPT perform on the United States medical licensing examination? the implications of large language models for medical education and knowledge assessment. JMIR Med Educ.

[2] Balel Y (2023). Can ChatGPT be used in oral and maxillofacial surgery?. J Stomatol Oral Maxillofac Surg.

[3] Eggmann F, Weiger R, Zitzmann NU, Blatz MB (2023). Implications of large language models such as ChatGPT for dental medicine. J Esthet Restor Dent.

[4] Strunga M, Urban R, Surovková J, Thurzo A (2023). Artificial intelligence systems assisting in the assessment of the course and retention of orthodontic treatment. Healthcare (Basel).

[5] Sabry Abdel-Messih M, Kamel Boulos MN (2023). ChatGPT in clinical toxicology. JMIR Med Educ.

[6] Huh S (2023). Are ChatGPT’s knowledge and interpretation ability comparable to those of medical students in Korea for taking a parasitology examination?: a descriptive study. J Educ Eval Health Prof.

[7] Lee H (2023). The rise of ChatGPT: exploring its potential in medical education. Anat Sci Educ.

[8] Schwendicke F, Samek W, Krois J (2020). Artificial intelligence in dentistry: chances and challenges. J Dent Res.

[9] Arif TB, Munaf U, Ul-Haque I (2023). The future of medical education and research: is ChatGPT a blessing or blight in disguise?. Med Educ Online.

[10] Nguyen TT, Larrivée N, Lee A, Bilaniuk O, Durand R (2021). Use of artificial intelligence in dentistry: current clinical trends and research advances. J Can Dent Assoc.

[11] Aminoshariae A, Kulild J, Nagendrababu V (2021). Artificial intelligence in endodontics: current applications and future directions. J Endod.

[12] Heo MS, Kim JE, Hwang JJ, Han SS, Kim JS, Yi WJ, Park IW (2021). Artificial intelligence in oral and maxillofacial radiology: what is currently possible?. Dentomaxillofac Radiol.

[13] Carrillo-Perez F, Pecho OE, Morales JC (2022). Applications of artificial intelligence in dentistry: a comprehensive review. J Esthet Restor Dent.

[14] Mohammad-Rahimi H, Rokhshad R, Bencharit S, Krois J, Schwendicke F (2023). Deep learning: a primer for dentists and dental researchers. J Dent.

[15] Temsah O, Khan SA, Chaiah Y (2023). Overview of early ChatGPT's presence in medical literature: insights from a hybrid literature review by ChatGPT and human experts. Cureus.

[16] Sallam M (2023). ChatGPT utility in healthcare education, research, and practice: systematic review on the promising perspectives and valid concerns. Healthcare (Basel).

[17] Zumsteg JM, Junn C (2023). Will ChatGPT match to your program?. Am J Phys Med Rehabil.

[18] Gupta R, Park JB, Bisht C (2023). Expanding cosmetic plastic surgery research using ChatGPT. Aesthet Surg J.

[19] Prada P, Perroud N, Thorens G (2023). Artificial intelligence and psychiatry: questions from psychiatrists to ChatGPT (Article in French). Rev Med Suisse.

[20] Mbakwe AB, Lourentzou I, Celi LA, Mechanic OJ, Dagan A (2023). ChatGPT passing USMLE shines a spotlight on the flaws of medical education. PLOS Digit Health.

[21] Sinha RK, Deb Roy A, Kumar N, Mondal H (2023). Applicability of ChatGPT in assisting to solve higher order problems in pathology. Cureus.

[22] Xue VW, Lei P, Cho WC (2023). The potential impact of ChatGPT in clinical and translational medicine. Clin Transl Med.

[23] DiGiorgio AM, Ehrenfeld JM (2023). Artificial intelligence in medicine & ChatGPT: de-tether the physician. J Med Syst.

[24] Biswas S (2023). ChatGPT and the future of medical writing. Radiology.

[25] Fatani B (2023). ChatGPT for future medical and dental research. Cureus.

[26] Kim SG (2023). Using ChatGPT for language editing in scientific articles. Maxillofac Plast Reconstr Surg.

[27] Cascella M, Montomoli J, Bellini V, Bignami E (2023). Evaluating the feasibility of ChatGPT in healthcare: an analysis of multiple clinical and research scenarios. J Med Syst.

[28] Alberts IL, Mercolli L, Pyka T, Prenosil G, Shi K, Rominger A, Afshar-Oromieh A (2023). Large language models (LLM) and ChatGPT: what will the impact on nuclear medicine be?. Eur J Nucl Med Mol Imaging.

[29] Anderson N, Belavy DL, Perle SM, Hendricks S, Hespanhol L, Verhagen E, Memon AR (2023). AI did not write this manuscript, or did it? Can we trick the AI text detector into generated texts? The potential future of ChatGPT and AI in Sports &amp; Exercise Medicine manuscript generation. BMJ Open Sport Exerc Med.

[30] Hopkins AM, Logan JM, Kichenadasse G, Sorich MJ (2023). Artificial intelligence chatbots will revolutionize how cancer patients access information: ChatGPT represents a paradigm-shift. JNCI Cancer Spectr.

[31] Mörch CM, Atsu S, Cai W (2021). Artificial intelligence and ethics in dentistry: a scoping review. J Dent Res.

[32] Ahmed N, Abbasi MS, Zuberi F, Qamar W, Halim MS, Maqsood A, Alam MK (2021). Artificial intelligence techniques: analysis, application, and outcome in dentistry-a systematic review. Biomed Res Int.

[33] Grischke J, Johannsmeier L, Eich L, Griga L, Haddadin S (2020). Dentronics: towards robotics and artificial intelligence in dentistry. Dent Mater.

[34] Shan T, Tay FR, Gu L (2021). Application of artificial intelligence in dentistry. J Dent Res.

[35] Arsiwala-Scheppach LT, Chaurasia A, Müller A, Krois J, Schwendicke F (2023). Machine learning in dentistry: a scoping review. J Clin Med.

[36] Ding H, Wu J, Zhao W, Matinlinna JP, Burrow MF, Tsoi JK (2023). Artificial intelligence in dentistry—a review. Front Dent Med.

[37] Ossowska A, Kusiak A, Świetlik D (2022). Artificial intelligence in dentistry-narrative review. Int J Environ Res Public Health.

[38] Kühnisch J, Meyer O, Hesenius M, Hickel R, Gruhn V (2022). Caries detection on intraoral images using artificial intelligence. J Dent Res.

[39] Revilla-León M, Gómez-Polo M, Vyas S, Barmak BA, Galluci GO, Att W, Krishnamurthy VR (2023). Artificial intelligence applications in implant dentistry: a systematic review. J Prosthet Dent.

[40] Bernauer SA, Zitzmann NU, Joda T (2021). The use and performance of artificial intelligence in prosthodontics: a systematic review. Sensors (Basel).

[41] Vishwanathaiah S, Fageeh HN, Khanagar SB, Maganur PC (2023). Artificial intelligence its uses and application in pediatric dentistry: a review. Biomedicines.

[42] Scott J, Biancardi AM, Jones O, Andrew D (2023). Artificial intelligence in periodontology: a scoping review. Dent J (Basel).

[43] Urban R, Haluzová S, Strunga M, Surovková J, Lifková M, Tomášik J, Thurzo A (2023). AI-assisted CBCT data management in modern dental practice: benefits, limitations and innovations. Electronics.

[44] Rokhshad R, Keyhan SO, Yousefi P (2023). Artificial intelligence applications and ethical challenges in oral and maxillo-facial cosmetic surgery: a narrative review. Maxillofac Plast Reconstr Surg.

[45] Vinayahalingam S, Berends B, Baan F, Moin DA, van Luijn R, Bergé S, Xi T (2023). Deep learning for automated segmentation of the temporomandibular joint. J Dent.

[46] Karobari MI, Adil AH, Basheer SN (2023). Evaluation of the diagnostic and prognostic accuracy of artificial intelligence in endodontic dentistry: a comprehensive review of literature. Comput Math Methods Med.

[47] Mahmood H, Shaban M, Rajpoot N, Khurram SA (2021). Artificial intelligence-based methods in head and neck cancer diagnosis: an overview. Br J Cancer.

[48] Eschert T, Schwendicke F, Krois J, Bohner L, Vinayahalingam S, Hanisch M (2022). A survey on the use of artificial intelligence by clinicians in dentistry and oral and maxillofacial surgery. Medicina (Kaunas).

[49] Huang H, Zheng O, Wang D (2023). ChatGPT for shaping the future of dentistry: the potential of multi-modal large language model. Arxiv.

[50] Ali K, Barhom N, Marino FT, Duggal M (2023). The thrills and chills of ChatGPT: implications for assessments in undergraduate dental education. Preprints.org.

[51] Agrawal P, Nikhade P (2022). Artificial intelligence in dentistry: past, present, and future. Cureus.

[52] Pahadia M (2023). ChatGPT in dentistry: is it worth the hype?. BDJ in Pract.

[53] Biswas S (2023). Role of ChatGPT in dental science. SSRN.

[54] Balaji SM (2022). Artificial intelligence and dental research. Indian J Dent Res.

[55] Homolak J (2023). Opportunities and risks of ChatGPT in medicine, science, and academic publishing: a modern Promethean dilemma. Croat Med J.

[56] Alkaissi H, McFarlane SI (2023). Artificial hallucinations in ChatGPT: implications in scientific writing. Cureus.

[57] Shen Y, Heacock L, Elias J, Hentel KD, Reig B, Shih G, Moy L (2023). ChatGPT and other large language models are double-edged swords. Radiology.

[58] Grünebaum A, Chervenak J, Pollet SL, Katz A, Chervenak FA (2023). The exciting potential for ChatGPT in obstetrics and gynecology. Am J Obstet Gynecol.

[59] Selvan P, Thavarajah R, Ranganathan K (2023). NLP and oral health information. J Oral Maxillofac Pathol.

[60] King MR (2023). The future of AI in medicine: a perspective from a Chatbot. Ann Biomed Eng.

[61] Howard A, Hope W, Gerada A (2023). ChatGPT and antimicrobial advice: the end of the consulting infection doctor. Lancet Infect Dis.

[62] Kurian N, Cherian JM, Sudharson NA, Varghese KG, Wadhwa S (2023). AI is now everywhere. Br Dent J.

[63] Mhlanga D (2023). Open AI in education, the responsible and ethical use of ChatGPT towards lifelong learning. Social Science Research Network.

[64] Johnson D, Goodman R, Patrinely J (2023). Assessing the accuracy and reliability of AI-generated medical responses: an evaluation of the Chat-GPT model. Res Sq.

[65] Baumgartner C (2023). The potential impact of ChatGPT in clinical and translational medicine. Clin Transl Med.

[66] Ali SR, Dobbs TD, Hutchings HA, Whitaker IS (2023). Using ChatGPT to write patient clinic letters. Lancet Digital Health.

